# A comprehensive review of methods based on deep learning for diabetes-related foot ulcers

**DOI:** 10.3389/fendo.2022.945020

**Published:** 2022-08-08

**Authors:** Jianglin Zhang, Yue Qiu, Li Peng, Qiuhong Zhou, Zheng Wang, Min Qi

**Affiliations:** ^1^ Department of Dermatology, Shenzhen Peoples Hospital, The Second Clinical Medica College, Jinan University, The First Affiliated Hospital, Southern University of Science and Technology, Shenzhen, China; ^2^ Dermatology Department of Xiangya Hospital, Central South University, Changsha, China; ^3^ School of Computer Science, Hunan First Normal University, Changsha, China; ^4^ Teaching and Research Section of Clinical Nursing, Xiangya Hospital of Central South University, Changsha, China; ^5^ Department of Plastic Surgery, Xiangya Hospital, Central South University, Changsha, China

**Keywords:** diabetic foot ulcer, medical image, deep learning, classification, object detection, semantic segmentation

## Abstract

**Background:**

Diabetes mellitus (DM) is a chronic disease with hyperglycemia. If not treated in time, it may lead to lower limb amputation. At the initial stage, the detection of diabetes-related foot ulcer (DFU) is very difficult. Deep learning has demonstrated state-of-the-art performance in various fields and has been used to analyze images of DFUs.

**Objective:**

This article reviewed current applications of deep learning to the early detection of DFU to avoid limb amputation or infection.

**Methods:**

Relevant literature on deep learning models, including in the classification, object detection, and semantic segmentation for images of DFU, published during the past 10 years, were analyzed.

**Results:**

Currently, the primary uses of deep learning in early DFU detection are related to different algorithms. For classification tasks, improved classification models were all based on convolutional neural networks (CNNs). The model with parallel convolutional layers based on GoogLeNet and the ensemble model outperformed the other models in classification accuracy. For object detection tasks, the models were based on architectures such as faster R-CNN, You-Only-Look-Once (YOLO) v3, YOLO v5, or EfficientDet. The refinements on YOLO v3 models achieved an accuracy of 91.95% and the model with an adaptive faster R-CNN architecture achieved a mean average precision (mAP) of 91.4%, which outperformed the other models. For semantic segmentation tasks, the models were based on architectures such as fully convolutional networks (FCNs), U-Net, V-Net, or SegNet. The model with U-Net outperformed the other models with an accuracy of 94.96%. Taking segmentation tasks as an example, the models were based on architectures such as mask R-CNN. The model with mask R-CNN obtained a precision value of 0.8632 and a mAP of 0.5084.

**Conclusion:**

Although current research is promising in the ability of deep learning to improve a patient’s quality of life, further research is required to better understand the mechanisms of deep learning for DFUs.

## 1 Introduction

Diabetes mellitus (DM) is a chronic disease due to impaired insulin secretion or insulin resistance or both (1). According to the International Diabetes Federation, the number of people with diabetes worldwide is 500 million in 2019 (1) and the number is expected to grow to 700 million adults by 2045 (2). Several complications associated with DM, including heart attack, stroke, blindness, kidney failure, and lower limb amputation (3) will increase mortality and decrease quality of life (2). About 19% to 34% of diabetic patients will develop diabetes-related foot ulcers (DFUs) (4). A person with DFU has a risk of poor wound healing. DFU may lead to lower limb amputation and may reduce survival rates (5). In addition, the most important risk factors involved in the development of foot ulcers in patients with diabetes are peripheral neuropathy and peripheral vascular disease (4).

With the development of artificial intelligence, artificial intelligence techniques have been applied to many medical images. Machine learning as a conventional artificial intelligence technique has become dominant for a long period. Here are some applications for the analysis of DFUs based on machine learning. Wang et al. (6) presented a cascaded two-stage classifier using support vector machines (SVMs) to determine the wound boundaries on foot-ulcer images, in which they extracted features from various colors and textures by using super pixels in the classifier training. Patel et al. (7) introduced a foot-ulcer detection system to recognize and classify DFUs into three categories, namely, granulation, slough, and necrosis by using a K means algorithm. They converted the color space from Red, Green and Blue (RGB) to Hue, Saturation and Intensity (HIS) and removed noise in image preprocessing. However, conventional machine-learning techniques have the following disadvantages: manual feature extraction is often affected by skin color and lighting and image resolution are less robust to combat the large change in normal and abnormal patterns in the population (8, 9). In addition, conventional machine-learning algorithms face many challenges such as the limitations of dealing with large image data, lack of sufficient domain knowledge, and having a multi-level abstract data representation (10).

Owing to the development of computer vision, deep-learning approaches demonstrated outstanding performance in image-processing tasks. Compared with conventional machine-learning algorithms, the advances in deep-learning approaches provided effective end-to-end automatic learning models from raw images. There are some reviews about applying deep-learning technology in medical-image analysis. Chan et al. (9) summarized medical-image analysis based on deep learning to aid diagnosis and face many related challenges. Hesamian et al. (11) summarized the achievements and challenges of medical-image segmentation by using deep-learning techniques. Cai et al. (12) wrote a review about the application of deep learning in medical-image classification and segmentation. These reviews extensively discussed the application of deep learning in various medical images, but none of the articles specifically reviewed the applications of deep-learning technology in the medical images of DFU. Yap et al. (13) summarized the object detection of DFUs for the Diabetic Foot Ulcers Grand Challenge (DFUC2020) data set with 2,000 images for training and 2,000 images for testing, but they only summarized the application for object detection of DFUs forDFUC2020. They did not mention the applications for classification, semantic segmentation, and instance segmentation for DFUs and object detection for DFUs for other data sets. Therefore, this review aims to understand and compare various deep-learning architectures for DFUs and the prediction accuracy of models established in various literature. This review will be analyzed from the following aspects: (1) popular deep-learning architectures used for image analysis, which focused on their pros and cons; (2) deep learning used in images of DFUs that included four applications: classification, object detection, semantic segmentation, and instance segmentation; (3) various types of challenges correlated with images of DFUs analyzed by using deep-learning techniques; and (4) conclusion and the future of deep learning.

## 2 Deep-learning techniques and application categories

**Figure 1:**
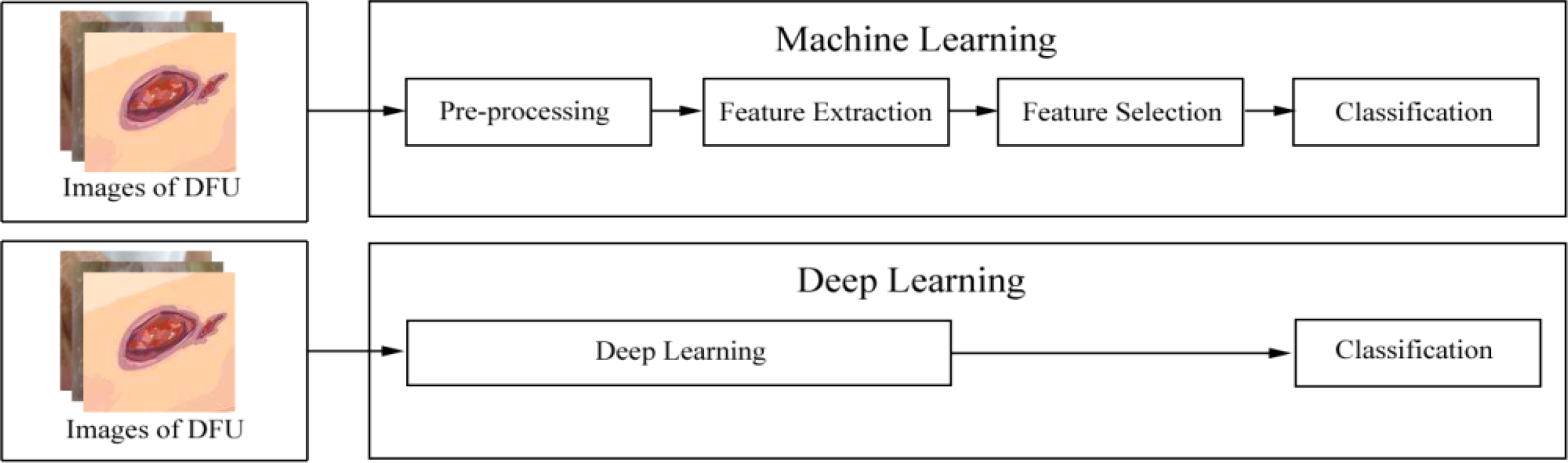
The difference between conventional machine learning and deep learning. After images of diabetes-related foot ulcer (DFU) are inputted into a machine-learning model, these images are processed following this pipeline: pre-processing, feature extraction, or feature selection. Then, these images are finally classified. However, after images of DFU are inputted into a deep-learning model, the model automatically learns representations of these images. Then, these images are finally classified. According to the Wagner–Meggitt (15) wound classification for foot-ulcer evaluation, the images of DFU was are considered grade 1 (superficial ulcer).

In recent years, deep learning, as a subset of machine learning, has seen a rapid development. Unlike conventional machine learning, which requires manual feature extraction and considers domain expertise, deep learning can automatically extract features with a change from hand-designed to data-driven features (14). The difference between conventional machine learning and deep learning in terms of extracting features is shown in **Figure 1**. Feature extraction in conventional machine learning often requires several processes such as pre-processing and feature extraction or feature selection. However, deep learning is often a computational model composed of multiple processing layers to automatically learn representations of data by transforming input information into multiple levels of abstraction (16) with simple but non-linear modules. By these transformations, deep-learning models will learn a very complex function. Importantly, because the learning process is automated, deep learning makes it easy to analyze thousands of cases that even human experts may not see and remember. As a result, deep learning can be more robust to a wide range of variations in features between different categories (9).

The applications of deep-learning technology are mainly divided into four categories, namely, classification, object detection, semantic segmentation, and instance segmentation (8, 17). These categories are illustrated in **Figure s1**. Classification is commonly used to identify the type of class of an object in an image or to provide a series of classes of objects in an image by their classification scores. Object detection in an image refers to classifying different objects and identifying the locations of classified image objects, which are marked by boundary boxes (18). Semantic segmentation consists of classifying each pixel of an image into an instance, where each instance corresponds to a class (19). Instance segmentation provides different labels for separate instances of objects belonging to the same object class (17).

## 3 Deep learning and classification in DFU images

This section discusses commonly used classification architectures of deep learning, including convolutional neural networks (CNNs) and deep convolutional neural networks (DCNNs). Then, a comprehensive description of deep learning in the classification of DFU images is introduced.

### 3.1 Deep-learning architectures of classification

Image classification, defined as the task of categorizing images into one of several predefined classes, is a fundamental problem in computer vision (20). CNNs, as one of the deep-learning architectures, have been widely used in image classification problems. CNN architectures originated from neural networks with a stack of layers to form a deep model (11, 20). Due to the advent of larger data sets and technological advances [the invention of graphics processing units (GPUs) and improved algorithms] (16, 19), DCNNs based on CNN architectures have become the most commonly used. In 2012, Krizhevsky et al. (21) proposed a DCNN called AlexNet containing five convolutional layers and three fully connected (FC) layers to classify approximately 1.2 million images into 1,000 classes. In 2014, Simonyan et al. (22) developed VGG16 (13 convolutional layers and 3 FC layers) and VGG19 (16 convolutional layers and 3 FC layers) that improved accuracy, respectively. In 2014, Szegedy et al. (23) proposed GoogLeNet with a module called inception and a 22-layer deep network to improve the utilization of computing resources. In 2015, He et al. (24) presented a residual network (ResNet) to solute degradation, improving the image classification effect by increasing network depth.

### 3.2 Overview of CNN architecture

**Figure 2:**
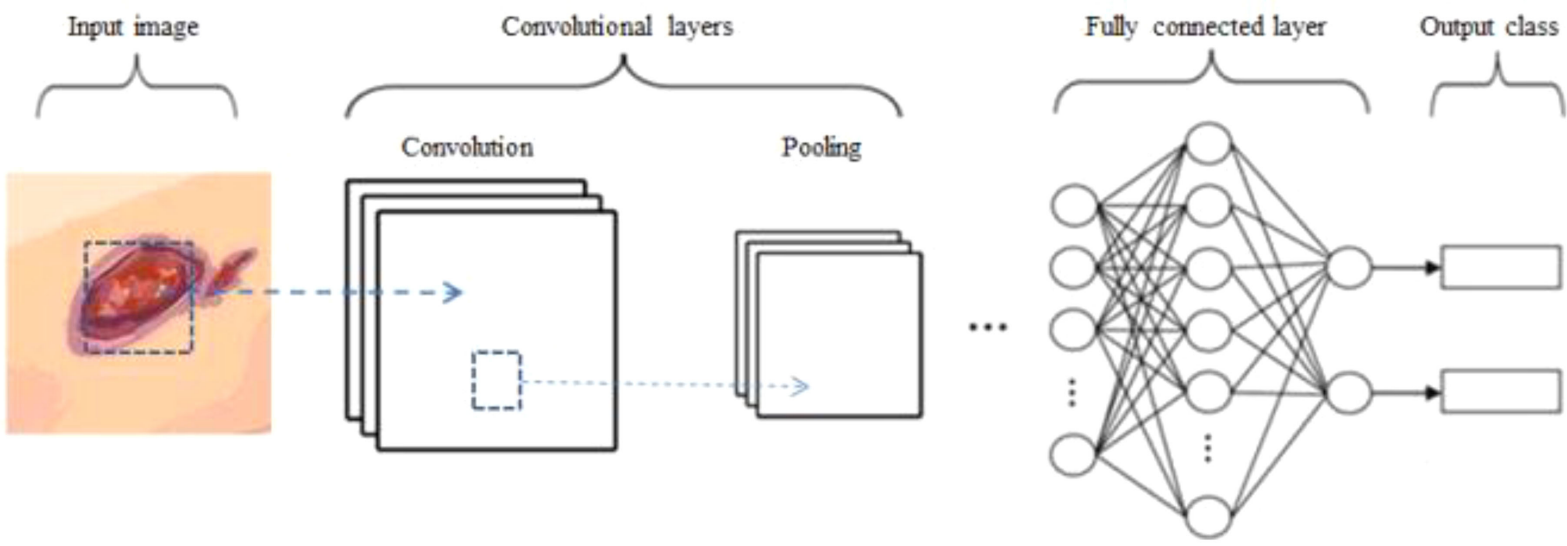
Architecture of a convolutional neural network (CNN) for image classification. Images of the DFU are inputted into the CNN model, which included convolution, pooling, dropout, and fully connected (FC) layers. After these images are processed by the model, they are finally classified.

CNNs have been used earlier in image classification. A typical CNN architecture consists of convolution, pooling, dropout, and FC layers (**Figure 2**). The convolutional layers learn the feature maps from input images as feature extractors (20). The different local matrices of the image are multiplied by a convolution kernel matrix, and then, they are added from the previous layer to the next layer as the convolved result (21). Non-linear features are extracted from the convolved results by the non-linear activation function such as sigmoid, tangent (Tanh), and rectified linear unit (ReLU). The non-linearity is used to adjust or cut off the generated output (25). The pooling layers are used to reduce the resolution and achieve invariance for the feature maps (20). They will avoid overfitting and reduce computational complexity. Pooling operations include max pooling, average pooling, and min pooling. The feature matrices in the last pooling layer are flattened and transformed into a vector to the first layer in the FC layer (21), and the number of FC layers is often more than one. The output of the last FC layer will be classified into a label by using the vector, which is flattened and transformed from the above feature matrices. If there are multiple classifications, the softmax will produce a distribution for more than one class labels (26).

### 3.3 Deep learning in the classification for images of DFU

**Figure 3:**
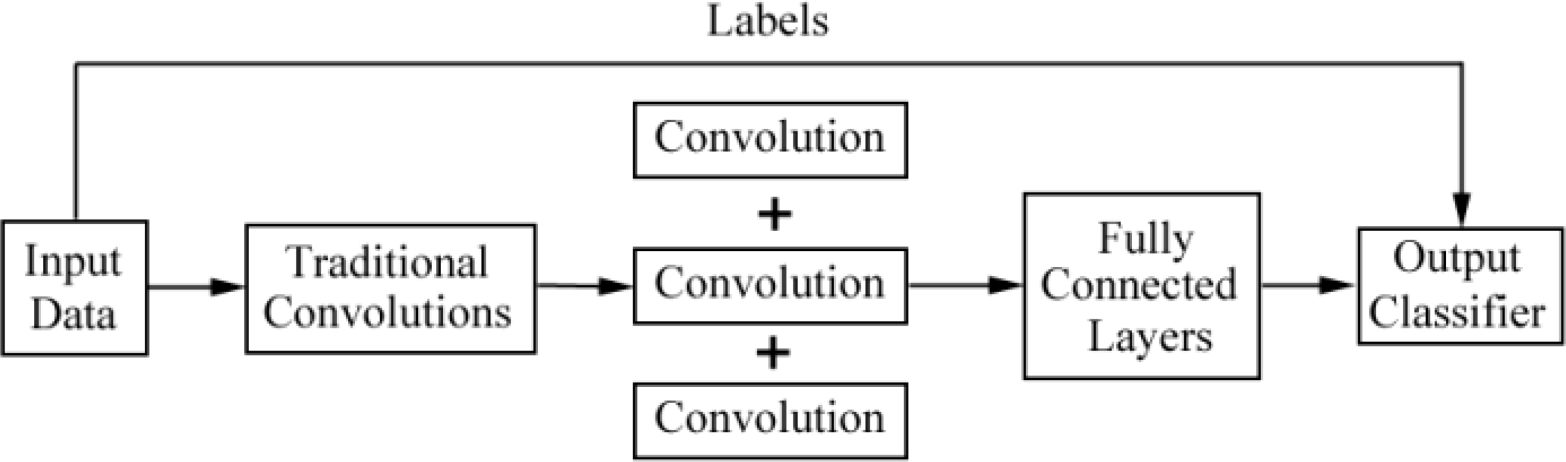
Overview of the architecture based on CNNs for DFU image classification in literature (10).

CNNs and their improved architectures as deep-learning techniques have been applied in the classification for images of DFU. Goyal et al. (10) used convolutional layers based on CNNs, called DFUNet, for the first time in DFU classification with 292 images. Traditional convolution layers with a single convolutional filter and parallel convolutional layers were used for the extraction of multiple features to classify two classes as normal skin (healthy skin) and abnormal skin (DFU). The architecture of DFUNet is shown in **Figure 3**. DFUNet achieved an area under curve (AUC) score of 0.961, in which these evaluation metrics outperformed those of the conventional machine learning and other deep-learning classifiers based on DCNNs such as LeNet, AlexNet, and GoogLeNet, because these DCNNs with more layers in a CNN do not lead to better performance or cause worse performance due to the gradient.

In 2020, Alzubaidi et al. (27) designed and implemented an architecture called DFU_QUTNet based on deep-convolutional neural network with 754 images. The improved CNN architecture included a series of layers such as input layer, convolutional layer, batch normalization (BN) layer, ReLU, an summation layer, average pooling layer, dropout layer, and FC layer. Their model increased the width and kept the depth of the network without drastically increasing its computational cost. The architecture of DFU_QUTNet is shown in **Figure s2**. The network was beneficial for gradient propagation and back propagation for the error. DFU_QUTNet was used to train SVM and K-nearest neighbor (KNN) classifiers to classify normal skin against abnormal skin (DFUs). Their model not only helped boost the details to learn and improve the extraction of major features but also handled many hard cases including small sizes of DFU, skin wrinkles, and patches with a toe. The DFU_QUTNet network outperforms DFUNet network (10), GoogleNet, VGG16, and AlexNet.

**Figure 4:**
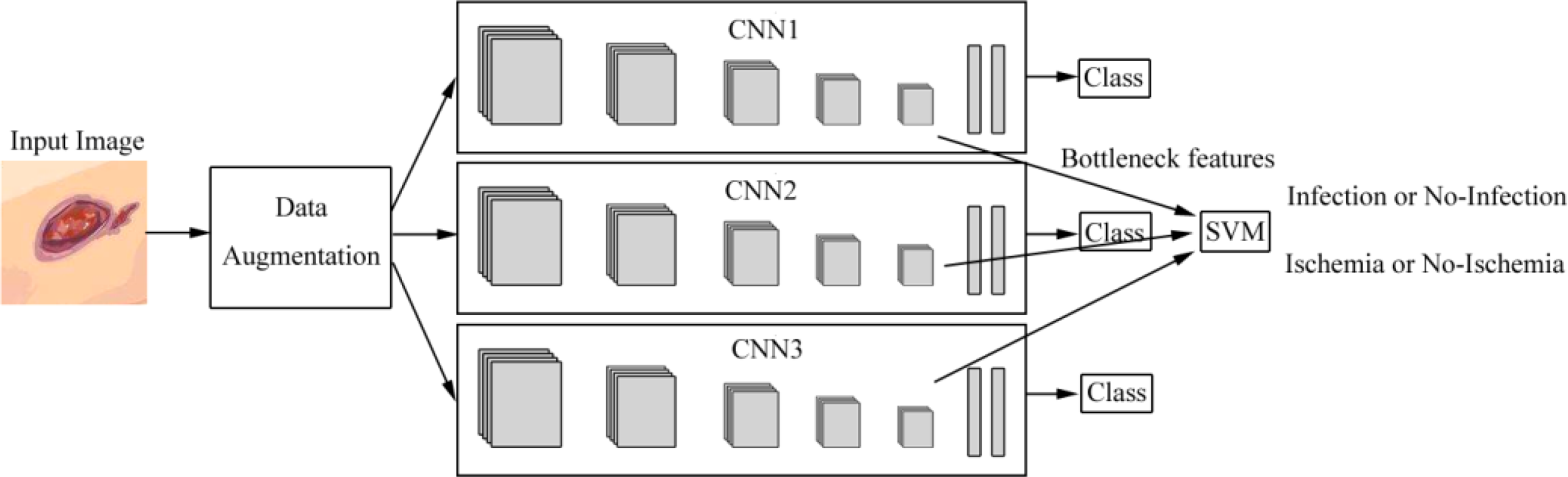
Overview of the architecture based on ensemble CNNs in literature (28). Features are extracted from CNNs and are fed into the support vector machine (SVM) classifier to perform the classification of infection or no infection, ischemia or no ischemia.

Goyal et al. (28) introduced the ensemble CNN model for binary classification to classify ischemia versus non-ischemia and infection versus non-infection with 1,459 images. The architecture is shown in **Figure 4**. They used hand-crafted super pixel color descriptors to extract the region of colors of interest from the images to improve visual cues for identifying ischemia and infection in DFUs. They also used a novel data-augmentation method based on faster R-CNN to avoid missing the regions of interest (ROIs) of the images. They proposed that the model performed better than the hand-crafted machine-learning algorithms for classification. The disadvantages of the model were that the number of ischemia and non-ischemia in DFU were unbalanced, the ground truth is labeled by visual inspection of experts and not supported by the medical notes or clinical tests, and the computer algorithm is not suitable for predicting the depth and the size of the wound based on non-standardized images.

Das et al. (29) introduced a DCNN based on ResKNet including a series of unique residual blocks of 2D convolution, batch normalization, and LeakyReLU with skip connections. For ischemia recognition of DFUs, they used four unique residual blocks (Res4Net) with 1,459 full foot images (210 ischemia and 1,249 non-ischemia). They achieved an AUC value of 0.9968. For infection recognition of DFUs, they used seven residual blocks (Res7Net) with 1,459 full foot images (628 infections and 831 non-infections). They achieved an AUC value of 0.8890.

Xu et al. (30) presented a model classifying DFUs based on a pre-trained vision transformer models with class knowledge banks (CKBs) as trainable units with 628 non-infection and 831 infection images of DFU, and 1,249 non-ischemia and 210 ischemia images of DFUs. The CKBs can extract and represent class knowledge to improve the performance of prediction with an accuracy of 90.90%. Cruz-Vega et al. (31) exhibited a model with fewer layers for 110 images compared with the 22 layers of GoogLeNet, which can classify diabetic foot thermograms into five categories. The model achieved some good results for some classes. For example, the model could well separate classes like 1-5, 2-5, 4-1, and so on. However, for neighboring classes, especially in classes like 3-4 and 4-5, the patterns were so similar and the training images did not provide sufficient information to learn in the model. It presented the lowest values in precision and accuracy. Wijesinghe et al. (32) presented an ensemble model based on DenseNet201, ResNet-18, and VGG-16 with 400 images, which was a mobile application with the best user performance.

The summary of deep learning in the classification for images of DFU is shown in **Table 1**. Although many architectures of DCNN based on CNN have been developed, whose performances are better than those of CNNs, we can see from the published papers that the ensemble models based on improved CNN architecture have better performance than many models based on a single DCNN. For example, Wijesinghe et al. (32) exhibited an ensemble model with an accuracy greater than 97% for the classification of diabetic foot thermograms. Goyal et al. (28) presented the ensemble model with a 90% accuracy in the ischemia classification.

**Table 1:** Summary of deep learning in the classification for images of diabetes-related foot ulcers.

Reference	Purpose	Network structure	Contributions	Limitations	Results
Goyal et al., 2018 (10)	Discriminating healthy and DFU	Parallel convolutions with a single filter	•Adopted for the first time •Better extraction	•Less automatic •Fewer images	•AUC: 0.961
Alzubaidi et al., 2019 (27)	Distinguishing healthy and DFU	Increasing DNN width with SVM and KNN as classifiers	•No computing increase •Increased accuracy •Better extraction •Handling small sizes	•Not mentioned	•Precision: 95.4% •Recall: 93.6% •F1-Score: 94.5%
Goyal et al., 2020 (28)	•Identifying non-ischemia and ischemia; •Identifying non-infection and infection	Ensemble CNN and SVM	•Improving identification •Avoid missing the region	•Data unbalance •Lacking depth and size	•Accuracy of ischemia: 90% •Accuracy of infection: 73%
Das et al., 2021 (29)	•Identifying non-ischemia and ischemia; •Identifying non-infection and infection	A DCNN based on ResKNet	•Achieving more than 95% in every evaluation metric in ischemia recognition •Improving its performance by increasing the number of residual blocks	•Not improving classification performance by further increasing the number of residual blocks	•AUC: 0.9968 for ischemia •AUC: 0.8890 for infection
Xu et al. 2022 (30)	•Identifying non-ischemia and ischemia; •Identifying non-infection and infection	A pre-trained vision transformer models with CKBs	•Improving the performance of DFU classifications	•Performance relies on the pre-trained network •Not considering the contrastive idea in samples	•Accuracy: 90.90 ± 1.74% •Sensitivity: 86.09 ± 2.98% •Precision: 95.00 ± 1.29% •Specificity: 95.59 ± 0.71% •F-measure: 90.30 ± 1.83% •AUC score: 96.80 ± 1.16%
Cruz- Vega et al., 2020 (31)	Discriminating diabetic foot thermograms	Shallow GoogLeNet	Multiple classes	Not easy to distinguish	•Sensitivity: 0.95 •Specificity: 0.94 •Accuracy: 0.94 •AUC: 0.95
Wijesinghe et al., 2019 (32)	The Wagner Ulcer Grading Scale using DNN	Ensemble model	•Best performance •Diabetic Retinopathy classification	No mention	Accuracy: >97%

## 4 Deep learning in the object detection for images of DFU

This section introduces commonly used object-detection architectures of deep learning. At present, popular deep-learning architectures of object detection include faster R–CNN (33), YOLO (34), EfficientDet (35), and SSD (36). We will discuss a comprehensive description of deep learning in object detection of DFU images.

### 4.1 Deep-learning architectures of object detection

**Figure 5:**
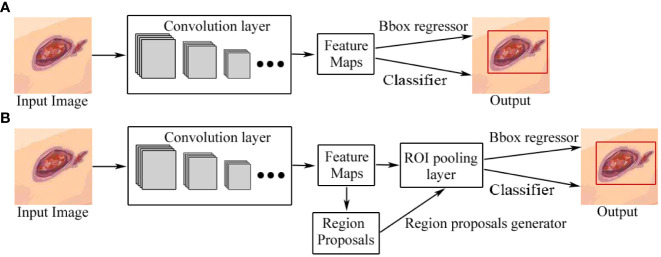
Two categories of object detection based on deep learning. **(A)** One-stage detection architecture. **(B)** Two-stage detection architecture. The difference between one-stage and two-stage models is that a two-stage model has a region-proposal process. Bbox regressor refers to the bounding box regressor.

Object detection in images refers to identifying the locations of objects and classifying the different objects contained in each image (37). At present, object detection based on deep learning can be divided into two categories: two-stage detection architecture and single-stage detection architecture. The former generates region proposals at first and then classifies each proposal into different object categories, and its advantage is accuracy, while the latter regards object detection as a regression or classification problem, adopting an integrated process to achieve final results (categories and locations) directly (18, 37), which are relatively fast but less accurate, compared with the former. The two categories of object detection based on deep learning are shown in **Figure 5**. Two-stage architectures include R-CNN ^40]^, fast R-CNN (38), faster R-CNN (33), and feature pyramid network (FPN) (39) while one-stage architectures include YOLO (34), EfficientDet (35), and SSD (36).

### 4.2 Overview of the faster R-CNN architecture

Faster R-CNN, introduced by Ren et al. in 2015, is an object-detection architecture with two stages (33). CNN architecture is the backbone of faster R-CNN, which generates feature maps by extracting features from the input image. The region proposal networks (RPNs) were introduced to take the convolution feature map as input, and then to output a series of proposing regions with objectness score generated by a sliding window convolution applied on the input feature map. The object detection network used in faster R-CNN is very much similar to that used in fast R-CNN. It is also compatible with VGG-16 as a backbone network. It also uses the region of interest (ROI) pooling layer for making a region proposal of fixed size and twin layers for the softmax classifier and the bounding box regressor, which is also used in the prediction of the object and its bounding box. In addition, the RPN and ROI pooling layer share the feature map, which reduces the number of parameters and prediction time. A typical architecture of faster R-CNN is shown in **Figure 5B**.

### 4.3 Object detection for images of DFU based on faster R–CNN

Compared with image classification, object detection includes the identification and location of the target (40). So far, there are few articles about deep learning in object detection for images of DFU based on faster R-CNN architecture. Da Costa et al. (41) proposed an adaptive faster R-CNN architecture with two main modules called the RPN and the classifier for the DFU detection. The architecture is shown in **Figure s3**. The feature maps extracted by ResNet-50 as the convolution layers serve as shared input for the RPN and the classifier. RPN suggests a set of rectangular object proposals with objectness score as input for the classifier, and each rectangular object proposal is classified with a score. The accuracy in the detection of small lesions was improved by adding the 64×64 anchor size to the set of standard anchor scales of faster R-CNN to maintain the original aspect ratios. The response time and precision were improved by using 100 ROI suggestions instead of 300 ROI suggestions as noted in a previous article. The detection time was reduced by sharing convolutional layers with the RPN as an advantage of the faster R-CNN. The model based on ResNet-50 had a better performance than that of the faster R-CNN with higher accuracy in the ROIs, a greater variety of ulcer formats detected, less false positives in the detection, and faster detection time. Compared with the SSD300 model, the model had higher precision and slower average speed.

**Figure 6:**
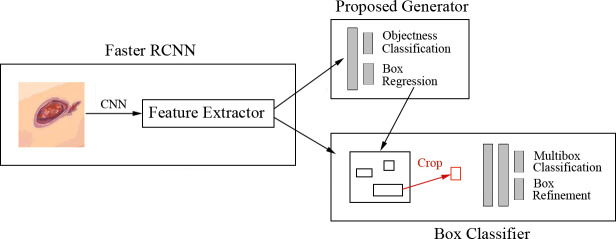
Faster R-CNN for DFU architecture (8) The model includes three stages. In stage 1, features are extracted by the CNN. In stage 2, region proposals are generated and refined by using the feature map extracted in stage 1. In stage 3, all the ROI boxes are classified and the bounding box regressor is used to refine the location of ROI boxes.

Goyal et al. (8) proposed a faster R-CNN with theInception-V2 model using two-tier transfer learning for DFU detection and localization with 1,775 images of DFU. The architecture is shown in **Figure 6**. Inception-V2 as a new iteration of GoogleNet reduced the computations and introduced a batch normalization layer to decrease the internal covariate shift and improve convergence. The model specifically matched the resource restrictions on mobile devices. The faster R-CNN with Inception-V2 is a multiple-stage model that needs to generate region proposals in advance, and then performs a fine-grained object detection. So, the disadvantage of the model is its slower speed than the SSD-MobileNet and SSD-InceptionV2 models, but faster R-CNN was introduced in this model due to the best trade-off between accuracy and speed. The model was trained on desktop computers with a GPU card and used on mobile devices due to the limited resources of a smartphone.

### 4.4 Overview of the YOLO detection architecture

Although faster R-CNN, as a two-stage architecture, is a popular technology due to its accuracy at present, its training needs more time for obtaining shared convolution parameters (37) and large resources (37) due to iterations through all the positions of the image by using a sliding window. To overcome the above disadvantages, the one-stage detection architectures extract features from the network to directly predict the classification and location of the object, which can reduce time consumption by generating bounding box and class probabilities. YOLO v1 model as You-Only-Look-Once (YOLO) was introduced by Redmon et al. (34) in 2016, which uses a single neural network for predicting bounding boxes and class probabilities as a regression problem. The model based on YOLO processes images faster than previous models. Because YOLO v1 makes more localization errors for small objects, YOLO v3 model was proposed by Redmon et al. in 2018, which improved speed and precision, compared with YOLO v1 (42). YOLO v5 is the latest in the YOLO series which is more flexible than the previous series of YOLO versions (42) and which uses a notable data augmentation called mosaic augmentation to detect smaller objects easier than that of previous YOLO versions (43).

### 4.5 Overview of the EfficientDet detection architecture

EfficientDet is the one-stage detection architecture proposed by Tan et al. (35) with optimizations. The model has EfficientNet as the backbone network. A weighted bi-directional feature pyramid network (BiFPN) as the feature network is used for easy and fast multi-scale feature fusion. EfficientDet scales the resolution, depth, and width with a compound scaling method. The EfficientDet architecture is shown in **Figure s4**.

### 4.6 Overview of the SSD detection architecture

Single-shot detector (SSD), introduced by Liu et al. (36), is a single-stage detection architecture for multiple categories without region-proposal generation and pixel or feature-resampling stages. The model generated scores for each object category and box offsets for a fixed set of default bounding boxes by using small convolutional filters, which are applied to feature maps. Additionally, the model produces predictions from multiple feature maps with different resolutions to achieve high detection accuracy.

### 4.7 Single-stage object detection architectures for DFU images

**Figure 7:**
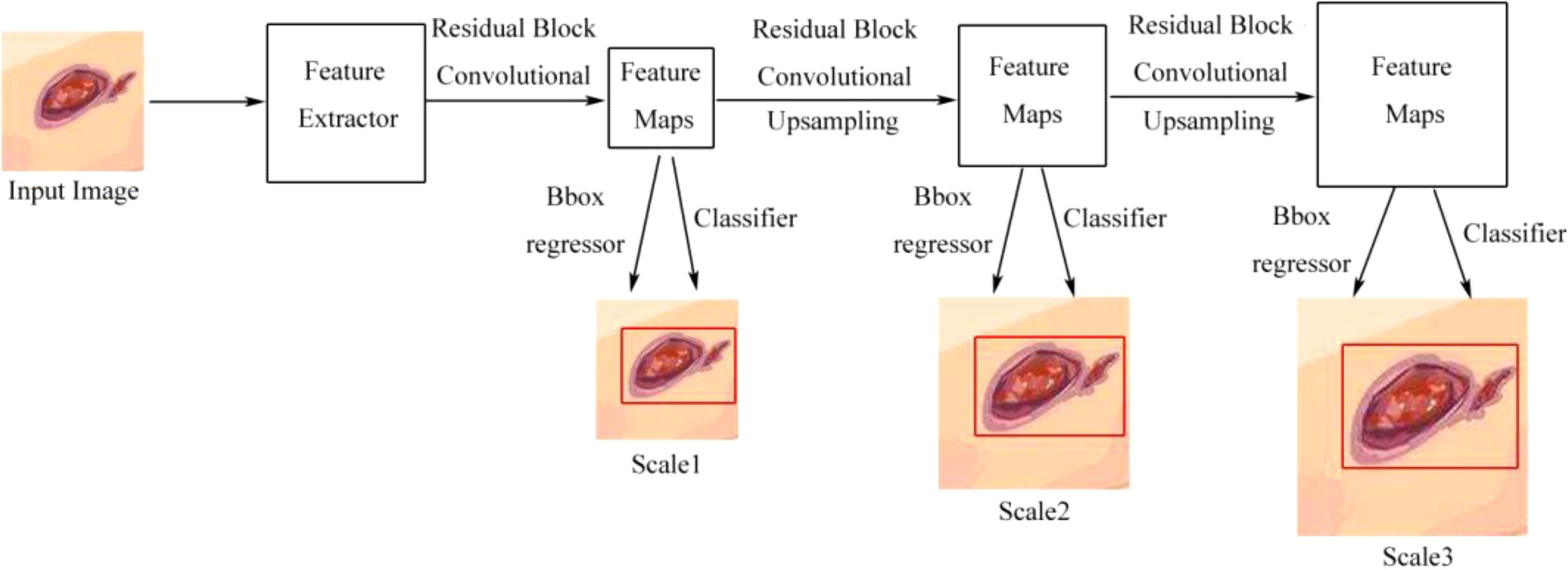
Detection flow chart of YOLO v3 without the region proposal process. Scale1, Scale2, and Scale3, respectively, represent the scale of detecting a small, medium, or large object (44).

Han et al. (44) used the real-time detection and location method for Wagner grades of DFU based on refinements on YOLO v3 as a single-stage model, which predicted classes and bounding-box regression simultaneously, without a region-proposal stage. The architecture is shown in **Figure 7**. Compared with the previous versions of YOLO v1, YOLO v3 has faster speed, better precision, and better object features by adjusting the network structure and adding a residual block. They presented the model in which several approaches were used to improve the performance including visually coherent image mix-up, classification head label smoothing, cosine learning rate, and common data-augmentation methods. The model achieved better speed and precision than SSD and faster R-CNN models and was usable in smartphone, so, the model achieved a good trade-off of speed and precision. The disadvantage of the model was that the accuracy of the averages for some categories was degraded due to the high inter-class similarity between Wagner grades, which led to misjudgment or missed judgment.

Goyal et al. (45) proposed a refined EfficientDet architecture for the detection of DFU images. To minimize false negative and false positive predictions, the architecture used a score threshold and removed overlapping bounding boxes. Their model, based on EfficientDet architecture, used a weighted bi-directional feature pyramid network (BiFPN) and a compound scaling method. These methods uniformly scaled the resolution, depth, and width so that the feature network, bounding box, and class prediction networks were finished simultaneously. They did not mention the performance of the model in their work.

Yap et al. (13) compared the deep learning-based algorithms for the detection and recognition of DFUs proposed by the winning teams of the Diabetic Foot Ulcers Grand Challenge (DFUC2020, with a comprehensive dataset consisting of 2,000 images for training and 2,000 images for testing), including faster R-CNN, three variants of faster R-CNN and an ensemble method, YOLO v3, YOLO v5, EfficientDet, and a new Cascade Attention Network. The best performance was from a variant of faster R-CNN called Deformable Convolution with a mean average precision (mAP) of 0.6940, an F1-Score of 0.7434, and the best recall value of 0.7687, while YOLO v5 achieved the lowest number of false positives and lower mAP and F1-Score. They found that these models had the following problems: many false positives for automatically localizing the ulcers and struggling to discriminate ulcers from other skin conditions.

The summary of deep learning in object detection for images of DFU is shown in **Table 2**. In practice, two-stage detection approaches with region proposal algorithms usually have a slightly better accuracy but are slower to run, while single-stage detection approaches are more efficient and do not have good accuracy as that of two-stage detection approaches.

**Table 2:** Summary of deep learning in object detection for images of DFU.

References	Purpose	Network structure	Contributions	Limitations	Results
Da Costa et al., 2021 (41)	DFU detection	•Adaptive faster R-CNN	•Better performance •Improving the accuracy of detecting small lesions	•Slower speed	•Precision: 91.4% •F1-score: 94.8%
Goyal et al., 2019 (8)	Detection and localization of DFU on mobile devices	•Faster R-CNN with InceptionV2 •Two-tier transfer learning	•Better performance •More accurate •Lightweight •Reducing computation •Decreasing internal covariate shift •Improving convergence	•Worse than R-FCNResnet101	•Precision: 91.8% •48 ms per image
Han et al., 2020 (44)	Real-time detection and location for the Wagner grades of DFUs	•Refined YOLO v3 •On smartphones	•Single-stage •Better acquisition of object features •Improving accuracy	•Inter-class similarity	•Accuracy:91.95% •Outperformed mAP •Good trade-off
Goyal et al., 2020 (45)	DFU detection	•Refined EfficientDet with distinct bounding boxes	•A weighted bi-directional feature pyramid network •Uniform scale •Minimizing false positives and false negatives	•No own data	•Without a report
Yap et al., 2020 *(* 13)	DFU detection	•An ensemble model	•A comprehensive evaluation •A variant of faster R-CNN with the best performance	•High false positives rate •Difficult to discriminate from other skin	•mAP: 0.6940 •F1-Score: 0.7434

## 5 Deep learning in the image segmentation for DFU images

This section discusses commonly used image segmentation architectures of deep learning, including the two categories, semantic segmentation and **instance segmentation.** Then, a comprehensive description of deep learning in the image segmentation for DFU images is introduced.

### 5.1 Semantic segmentation

Semantic image segmentation is a process where each pixel of an image is labeled with the class of its enclosing object without differentiating object instances (17). In other words, semantic segmentation deals with the identification/classification of similar objects as a single class from the pixel level. Current popular architectures for semantic image segmentation include FCN (46), SegNet (47), and U-Net (48).

### 5.2 Overview of the fully convolutional network (FCN) architecture

An FCN based on CNN was proposed by Long et al. (46) who adopted previous models for classification such as AlexNet, VGG net, and GoogLeNet by replacing previous FC layers in CNNs with a fully convolutional layer for semantic segmentation. FCNs usually use downsampling and upsampling. In the first half of the model, the spatial resolution of the image is downsampled to develop complex feature mappings. With each convolution, finer information of the image is captured. At this stage, highly efficient discrimination between different classes is obtained; however, the location information is lost. To recover the location information, downsampling is followed by an upsampling procedure, which takes multiple lower resolution images as input and gives a high-resolution segmentation map as output, with each pixel classified into the highest probability class. The FCN architecture is shown in **Figure s5**.

### 5.3 Overview of the U-Net architecture

**Figure 8:**
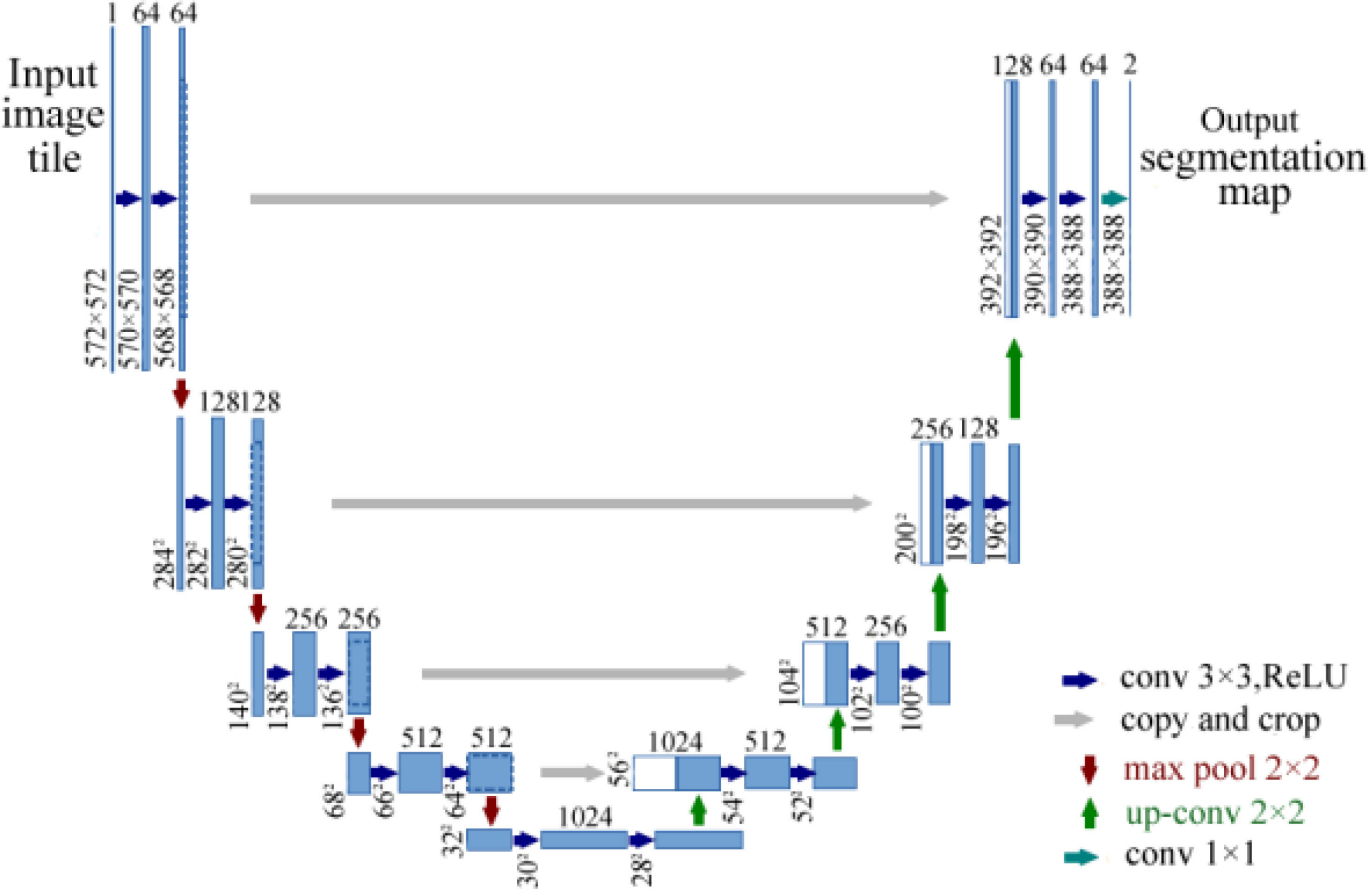
Architecture of U-Net for semantic segmentation (48). The model consists of a contracting path and an expansive path.

U-Net based on FCN was designed by Ronneberger et al. (48). U-Net is a common and successful algorithm used in semantic segmentation for images. The architecture of FCN includes an FC layer at the end, while U-Net simply applies convolutional layers. U-Net is a perfectly symmetric architecture with a U shape and consists of two paths, namely, a contracting path and an expansive path. The contracting path is a typical architecture of a convolutional network, which produces a low-dimensional representation to obtain feature extractions. The expansive path increases the resolution of the output by upsampling the various feature maps from the contracting path. The architecture of U-Net is shown in **Figure 8**. U-Net is very useful in dense prediction tasks, in which each pixel must be labeled (so-called semantic segmentation), and it is able to converge with few training samples (48).

### 5.4 Instance segmentation

Instance segmentation is able to deal with the correct detection of all objects in an image and provides different labels for different instances of the same class, which combines object detection and semantic segmentation simultaneously (17). An example of a neural network that is used for instance segmentation is mask R-CNN (49).

### 5.5 Overview of the mask R-CNN architecture

Mask R-CNN was presented by He et al. (49) for object instance segmentation in 2017, which was based on faster R-CNN. In the first part of mask R-CNN, ROIs are selected. An ROI is a patch of the input image that contains an object with high probability. Multiple ROIs are identified for each input image. In the second part of mask R-CNN, shown in **Figure s6**, each ROI is able to obtain three model outputs simultaneously: a class label and a bounding box for each candidate object from faster R-CNN, and the object mask to extract a finer spatial layout of an object from the addition of a third branch of mask R-CNN. Therefore, mask R-CNN is an extension of faster R-CNN and works by adding a branch for predicting an object mask (ROIs) in parallel with the existing branch for bounding-box recognition.

### 5.6 Deep learning in the semantic segmentation for DFU images

There are few articles about deep learning in the semantic segmentation and instance segmentation for images of DFU.

**Figure 9:**
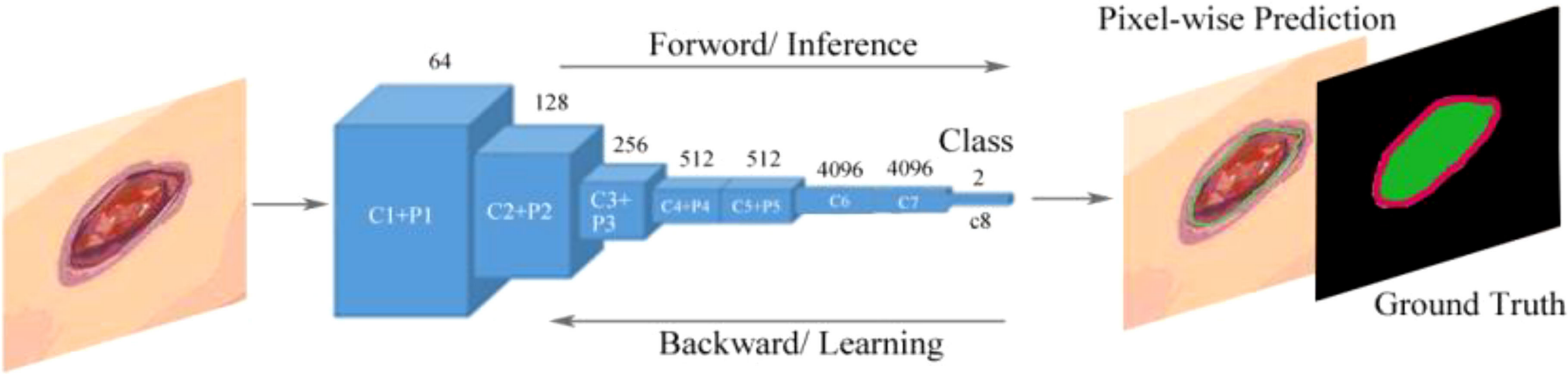
Fully convolutional networks (FCNs) for the semantic segmentation of DFUs (50). The model learns features with forward and backward learning for segmentation. C1–C8 are convolutional layers and P1–P5 are max-pooling layers.

Goyal et al. (50) proposed a two-tier transfer learning to train the FCNs to automatically segment the ulcer and surrounding skin. They used three models, namely, FCN-32s, FCN-16s, and FCN-8s based on the VGG-16 network and one model called FCN-AlexNet for segmenting images of DFUs. They found that these models can retrieve feature hierarchies. FCN architectures with two-tier transfer learning performed more effective pixel-wise segmentations on DFU data sets and achieved better convergence of weights than random initialization of weights for all layers of network. The FCN-16s and FCN-8s models can produce more irregular contours of both DFU and surrounding skin, but the FCN-AlexNet and FCN-32s models struggled to draw irregular boundaries to perform accurate segmentation because these models were not able to detect the small DFU and to distinguish surrounding skin or to detect very small parts of them. FCN-16s was the best performer and FCN-AlexNet was the worst performer among all the FCN architectures. The overview of the FCN architecture is shown in **Figure 9**.

Rania et al. (51) performed segmentation using U-Net for DFUs. A smartphone and a small thermal camera were used to obtain images with thermal information in which temperature indicators from the thermal image can help detect tissue infection and inflammation. Then, 112 DFU images were resized to a resolution of 512 × 512 pixels and 92 images were used to train the U-Net model, while 22 images were used for validation, which was based on using the Keras framework with the TensorFlow backend. The U-Net model could generate a mask similar to the ground truth and correctly segment the ulcer area using few images, demonstrating precise DFU segmentation by automatically calculating the ulcer area after segmentation and carrying out wound tissue analysis based on color and temperature.

**Figure 10:**
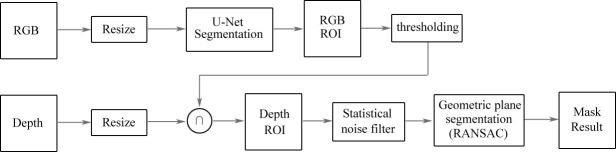
Architecture based on U-Net for the automatic segmentation of DFUs (52). Images with RGB information are inputted into the U-Net model to obtain the ROI. Then, the ROI is set on the depth image. After a second segmentation is applied to extract geometric models, the results of semantic segmentation are obtained from the model.

Hernández et al. (52) presented a model of automatic segmentation based on U-Net architecture using multimodal images to identify and delineate images of feet. The architecture is shown in **Figure 10**. They obtained a foot image with both RGB and depth information, and of the thermal infrared images including 59 images, 30 images were employed for training the supervised algorithm and 29 images were used for testing. Then, they compared the temperature of two foot images of a patient to monitor DFUs. The architecture was based on U-Net to perform an automatic segmentation of the feet with the RGB information of the images, and the depth information of the image was used to improve the segmentation results provided by U-Net by a segmentation of planes in the depth image. The segmentation results were improved by an approach called RANdom SAmple Consensus (RANSAC) for searching the best segmentation of planes in images with the depth information. The model also demonstrated a great performance by using fine-tuning for the encoder path using a small training data set, and could provide automatic segmentation results in a short time. The model achieved a better performance than other traditional segmentation methods and a basic U-Net segmentation system.

**Figure 11:**
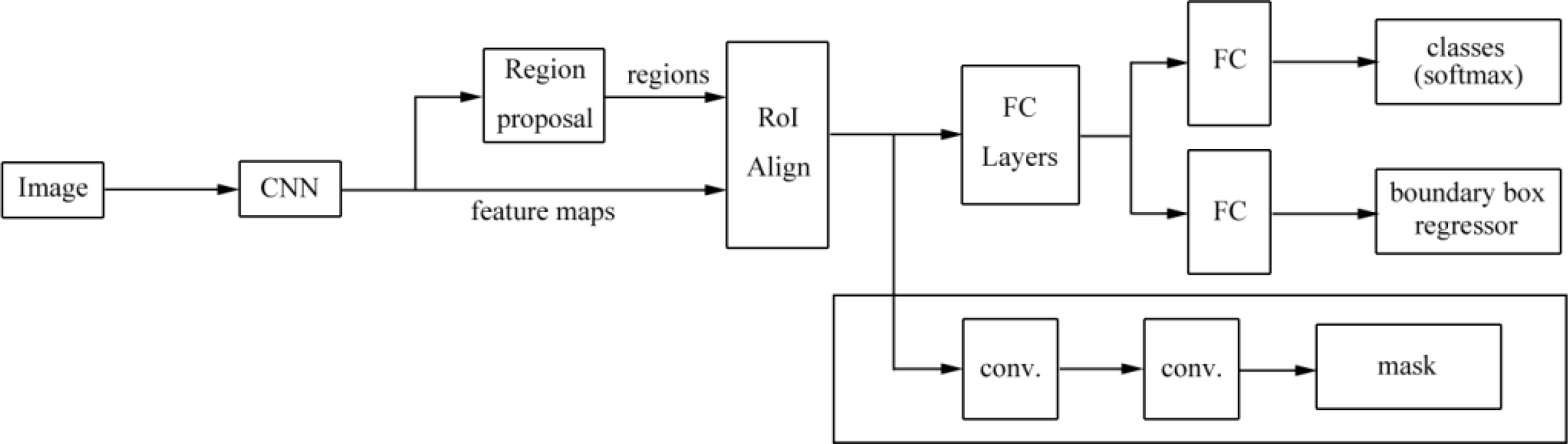
The mask R-CNN architecture proposed by Gamage et al. (53). The model is a region proposal network with two outputs (a class label and an object region). Mask R-CNN outputs an object mask, ROI boxes, and a bounding box regressor. This mask supports object segmentation more accurately.

Gamage et al. (53) proposed a mask R-CNN model that automated locating and segmenting ulcer boundaries for diabetic patients. The model extended the faster R-CNN architecture and is shown in **Figure 11**. Firstly, region proposals were generated with two different backbone CNNs, namely, ResNet-50 and ResNet-101, and the features were extracted from the backbone network, in which low level features were extracted in the early layers of the network and object instances were detected in the later layers of the network. Then, ROIs and bounding boxes were generated by a region proposal network. The ROIs were classified by the classifier and bounding boxes were refined by the bounding-box regressor. Finally, a mask was generated for the classified ROIs. The model can be used in object detection, localization, and instance segmentation of images of ulcer. The mask R-CNN model obtained a higher accuracy and performed better than those of U-Net models and can replace manual measurements of ulcers.

Zhao et al. (54) introduced an intelligent measurement model for DFUs based on mask R-CNN and RetinaNet with 1,042 images of DFUs, in which mask R-CNN was used for the ulcer tissue color instance segmentation and RetinaNet was used for the digital scale target detection. The mAPs of the color region segmentation were 87.9% and 63.9% for the training set and the test set, respectively. The mAPs of the ruler scale digital detection were 96.5% and 83.4% for the training set and the test set, respectively, and the average error of the intelligent measurement result was about 3 mm, which compared with the manual measurement of DFUs. The summary of deep learning in the semantic segmentation for images of DFU is provided in **Table 3**.

**Table 3:** Summary of deep learning in image segmentation for images of DFU.

References	Purpose	Network structure	Contributions	Limitations	Results
Goyal et al., 2017 (50)	•Automatic segmentation	•Two-tier transfer learning with three models	•Obtaining pixel-wise prediction •Better convergence •Retrieving feature hierarchies •Producing irregular contours	•Issues of small size and part •Accuracy of irregular boundaries •Some similar tissues of DFU and surrounding skin	•Dice (ulcer): 0.794 ± 0.104 •Dice (surrounding): 0.851 ± 0.148 •Combination: 0.899 ± 0.072
Rania et al., 2020 (51)	•Semantic segmentation	•U-Net •V-Net •SegNet	•Superior segmentation	•Fewer images	•Accuracy: 94.96% •IoU: 94.86% •DSC: 97.25%
Hernández et al., 2019 (52)	•A monitoring system for automatic segmentation with multimodal images	•No FC layers based on U-Net	•Great performance •Segmentation enhancement •Plane segmentation with RANSAC	•Fewer images	•Short time •Better performance
Gamage et al., 2019 (53)	•Automatic detection of location and segmentation of ulcer boundaries	•Mask R-CNN and ResNet-50 •Mask R-CNN and ResNet-101	•Object detection, localization, and instance segmentation •High accuracy and performance	•Not mentioned	•Precision: 0.8632 •mAP: 0.5084
Zhao et al., 2021 (54)	•An intelligent measurement model for DFUs	•Mask R-CNN •RetinaNet	•Instance segmentation of ulcers •Digital scale target detection •High accuracy compared with the manual measurement of DFUs	•Not mentioned	•mAP of the region of segmentation: 63.9% •mAP of the ruler scale digital detection: 83.4%

## 6 Performance evaluation

### 6.1 Performance evaluation metrics

Performance evaluation metrics are used to evaluate the quality of machine-learning algorithms, and this is also true in deep-learning algorithms. There are many different performance evaluation metrics for a deep-learning model, which can be often combined to evaluate a model. Moreover, the correct use of performance evaluation metrics is a key factor showing whether the model is working properly and whether it works in the best way (26). Therefore, in this section, the performance evaluation metrics used in the research of the above literature on deep-learning models in DFUs are described in **Table 4** to help other researchers make better choices on deep-learning models.

**Table 4:** Performance evaluation metrics used in the research of the above literature for deep learning in DFUs.

Evaluation metrics	Formula and the source literature	Source references
Accuracy	$\text{Accuracy}=\frac{TN+TP}{TN+TP+FN+FP}$ (35)	(3, 28–30, 43, 50)
Sensitivity	Sensitivity=TP/(TP+FN) (35)	(29, 30)
Specificity	Sensitivity=TN/(TN+FP) (35)	(29, 30)
Precision	Precision=TP/(FP+TP) (35)	(8, 27, 29, 39, 51)
Recall	Recall=TP/(TP+FN) (35)	(27)
AUC	$\text{AUC}=\frac{\sum_{ins_{i}\in postiveclass}rank_{ins_{i}}-\frac{M\times \left(M+1\right)}{2}}{M\times N}$ (35)	(10, 29, 30)
F1-Score	$\text{F Score}=\text{F Score}=\frac{2\times Recall\times Precision}{Recall+Precision}$ (35)	(27, 28, 39, 45)
Average precision (AP)	$\text{AP}=\frac{\sum_{q=1}^{Q}\frac{TP_{i}}{TP_{i}+FP_{i}}}{Q}$ (35)	
Mean average precision (mAP)	$\text{mAP}=\frac{\sum_{q=1}^{Q}AveP\left(q\right)}{Q}$ (35)	(43, 45, 51, 53)
Dice similarity coefficient (DSC)	$\text{DSC}=\frac{2\times TP}{2\times TP+FP+FN}$ (50)	(50)
Union index (IoU)	$\text{IoU}=\frac{TP}{TP+FP+FN}$ (50)	(50)

Confusion matrix contains information about actual and predicted classifications in deep-learning models. Confusion matrix is given in **Table S1**. Some conceptions in confusion matrix are defined as follows: If a deep-learning model correctly predicts the positive class, it is a true positive (TP), otherwise, it is a false positive (FP). If a deep-learning model correctly predicts the negative class, it is true negative (TN), otherwise, it is false negative (FN). These conceptions are used in the performance evaluation metrics of deep-learning models for DFUs.

### 6.2 Improving performance

There are two methods to improve performance for deep-learning models of the DFU analysis, namely, dropout and transfer learning.

#### 6.2.1 Dropout

Deep-learning models based on deep neural networks with many parameters have two disadvantages: Deep neural networks with several non-linear hidden layers and limited training data can learn complicated relationships and result in overfitting. Moreover, a combination of machine-learning models can improve performance, but it is expensive for different architectures of models to be trained on different data. Therefore, it is difficult to overcome overfitting by combining many different models at test time (55).

To solve the above issues, dropout, as a technique similar to regularization, is presented. It can reduce the risk of overfitting and efficiently combine many different neural network architectures (55). In the dropout concept, the neurons are randomly turned on and off in each layer from the deep-learning network during training (27), that is to say, by dropout, the neurons are temporarily removed from the network with their incoming and outgoing connections. A dropout neural net model is shown in **Figure s7** (55). Dropout can improve the performance of neural networks on a range of benchmark data sets.

#### 6.2.2 Transfer learning

Transfer learning can recognize and apply knowledge and skills learned in previous tasks to a novel task. Transfer learning can be a powerful tool to enable training a large target network without overfitting (56) and has better performance (11). Alzubaidi et al. (27) have used three pre-trained CNN models, namely, GoogleNet, AlexNet, and VGG16 by training and testing image data sets such as ImageNet, and these models have been fine-tuned on medical image data sets. Pre-trained models enhance performance by using transfer learning. Goyal et al. (50) used two-tier transfer learning to perform more effective segmentation on a DFU data set. In a first-tier transfer learning, relevant CNN models are trained on the ImageNet data set (20). In a second-tier transfer learning, the models trained on the Pascal Visual Object Challenge (VOC) segmentation data set. These pre-trained models are used for training on DFU data sets for better convergence of weights rather than random initialization of weights. The two-tier transfer learning is shown in **Figure s8** (50).

## 7 Challenges

### 7.1 Smaller data set

A typical deep-learning framework is often composed of multiple neural network layers, thus, many parameters need to be set and optimized. If there are few medical images in the training data set and many parameters need to be optimized in the deep-learning model, it will cause overfitting (11). Because collecting medical images is a time-consuming and laborious task, the articles we found used as few as 59 DFU images and as many as 4,500 DFU images, and most of the articles used hundreds and a few of them used more than 2,000 DFU images. Although there is no hard requirement for the minimum training data set size, the general experience is to have at least about 10 times the number of samples as the number of parameters in the network (57). In addition, if the training data set cannot represent the characteristics of real patients, the deep-learning model is unlikely to obtain accurate results. These challenges could be solved in the following ways: Try to collect more medical image data; use more kinds of image-capture equipment; make the data sets more diverse; and adopt data augmentation.

### 7.2 Limited annotated data

Semi-supervised learning (SSL) provides a powerful framework for leveraging unlabeled data when labels are limited or expensive to obtain.

Deep-learning techniques usually need DFU images annotated by a podiatrist specializing in the diabetic foot. Goyal et al. (50) created ground truth for each image with DFU by using the annotator by Hewitt et al. (10) and exporting the output to an extensible markup language (XML) file. Deep-learning models need a large number of annotated images to train the models, and it is a laborious task to annotate a large number of images. Oliver et al. (58) presented SSL. Its goal is for a small number of labeled samples to be propagated to other unlabeled data. The challenge of limited annotated data is solved by SSL as it allows the classifier to achieve higher accuracy faster while reducing the number of annotated samples.

### 7.3 Choosing the right deep-learning architecture and hyperparameters

Different deep-learning architectures have different advantages and disadvantages, and different deep-learning architectures will be selected according to the characteristics of the input data and research purposes. The analysis of medical images of DFU can be divided into classification, object detection, and semantic segmentation. For example, CNNs are suitable for classification, faster R-CNNs are suitable for object detection, and FCNs are suitable for semantic segmentation. At present, choosing the right deep-learning architecture is a challenging task, and more models and algorithms need to be tried and further studied in the future.

When a deep-learning architecture is chosen, a large number of hyperparameters will be set and optimized by training the deep-learning model, in which hyperparameters are automatically set to optimize performance and reduce human effort (59). At present, automatically optimizing hyperparameters usually uses a random search, such as Bayesian optimization (60).

### 7.4 Changing the black box into a white box

Although deep-learning models have achieved good results in many domains, a deep-learning model is still a black box and lacks an explanation of its internal mechanism, which makes it difficult for clinicians to understand its results. It is very important for the interpretability of medical image analysis systems based on deep learning. It can help doctors understand the disease and make the correct diagnosis for the benefit of the patients. Xiang et al. (61) presented an interpretable model called local interpretable model-agnostic explanations (LIME) which was applied to extract evidence from the skin images to support the classification results by visualizing models. Wulczyn et al. (62) introduced a deep-learning-based image-similarity model that generated human-interpretable histologic features by clustering embeddings. The model could explain majority of the variance. Wu et al. (63) used a model for finding interpretable representations that can explain medical imaging predictions. At present, although some models have achieved some interpretable visualization results, some researchers thought these results are far from sufficient and some explanations are even unreliable. It is believed that the transition from black box to white box is still in the early stages of research.

## 8 Conclusion and future trends

Current deep learning has been successfully applied in classification, target detection, and segmentation for medical images. With the technology developed, more multimodal data can be collected. These data include medical images (X-ray, CT, MRI, PET, etc.) and other forms of medical resources (electronic medical records, genomics, bioinformatics, drug responses, etc.). The variety of data is so complicated that more advanced deep-learning architectures need to be developed.

Deep learning has been widely used in medicine to solve problems such as disease diagnosis, prediction, medical-image classification, detection, and segmentation. The occurrence and development of diseases often include multiple stages, and how to better apply deep-learning models to all stages of medical diagnosis and treatment has become more challenging and need three aspects: technological advancement, more data collection, and more medical experience.

At present, a number of excellent algorithms have been used in medical domain, but setting deep-learning model parameters and training data need more time; therefore, it is necessary to accelerate the development of deep-learning models, improve deep learning algorithm, and manufacture better and faster hardware. We should improve the efficiency and accuracy of algorithms by improving them or combining multiple architectures. Furthermore, we should exploit approaches such as using a graphic processing unit, large-scale clusters of machines in a distributed environment, and a cloud computing platform. Despite challenges such as small data sets, limited annotations, lack of interpretability, and time-consuming training, deep-learning technology will have a huge impact on medicine and will benefit medicine, doctors, and patients.

## Author contributions

JZ and YQ performed conceptualization, methodology, writing original draft, formal analysis, and final approval of the version to be submitted. LP performed investigation and revision; ZW carried out project administration, reviewing the final draft and editing, and final approval of the version to be submitted. MQ carried out investigation, visualization, and final approval of the version to be submitted. QZ was involved in supervision, validation, and final approval of the version to be submitted. All authors contributed to the article and approved the submitted version.

## Funding

This work was supported by the National Natural Science Foundation of China (under Grants 82073018, 82073019) and Shenzhen Science and Technology Innovation Committee (JCYJ20210324114212035), and also funded by Hunan province nature science foundation of China(under Grant 2022JJ30189), and Teaching Reform Research Project of Universities in Hunan Province(under Grant HNJG-2021-1120).

## Conflict of interest

The authors declare that the research was conducted in the absence of any commercial or financial relationships that could be construed as a potential conflict of interest.

## Publisher’s note

All claims expressed in this article are solely those of the authors and do not necessarily represent those of their affiliated organizations, or those of the publisher, the editors and the reviewers. Any product that may be evaluated in this article, or claim that may be made by its manufacturer, is not guaranteed or endorsed by the publisher.
