# Supplementary material for: A comprehensive review of methods based on deep learning for diabetes-related foot ulcers
Source: Front Endocrinol (Lausanne). 2022 Aug 8;13:945020. doi: 10.3389/fendo.2022.945020 (PMC9394750; doi:10.3389/fendo.2022.945020)
Supplement: Supplementary file 1 [file DataSheet_1.docx]

Appendix


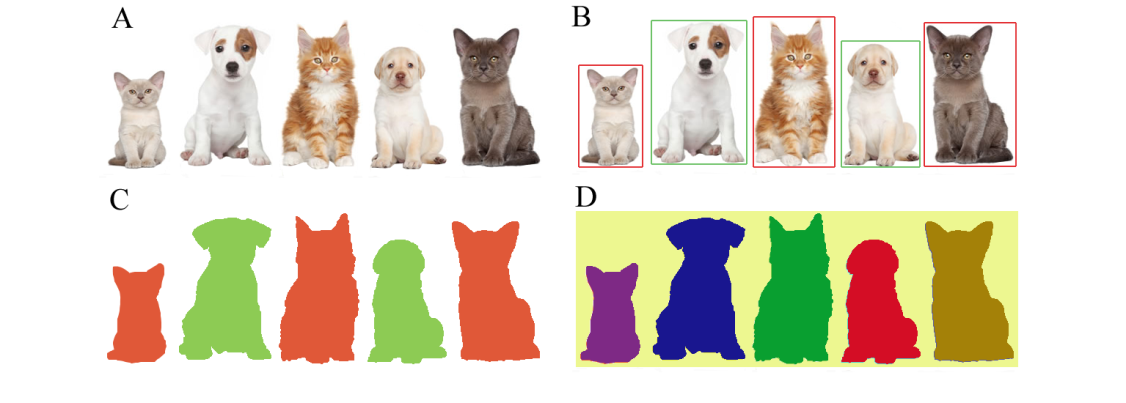


**Fig.s1 Four categories for inspection of an image A. Image** **Classification, B.** **Object detection C.** **Semantic segmentation, D.** **Instance segmentation**.


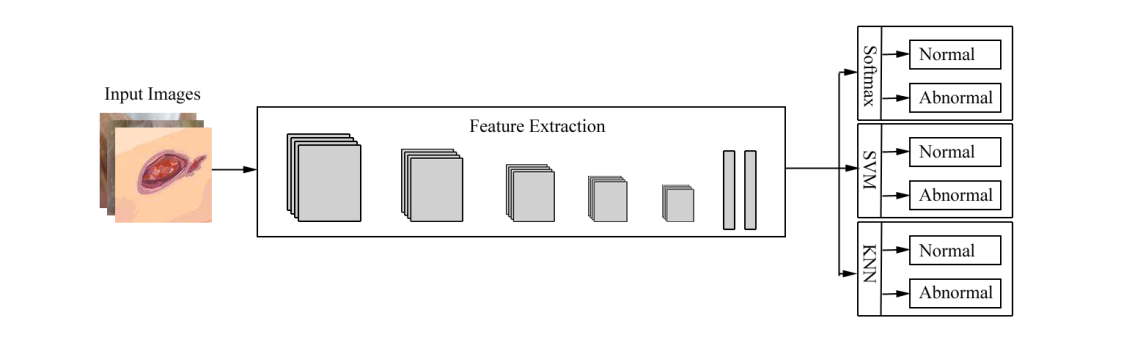


**Fig. s2 An overview of the architecture in literature ^[27]^** Features were extracted from the improved CNNs and were fed into Softmax, SVM or KNN classifier to perform classification of healthy feet or DFU, respectively.


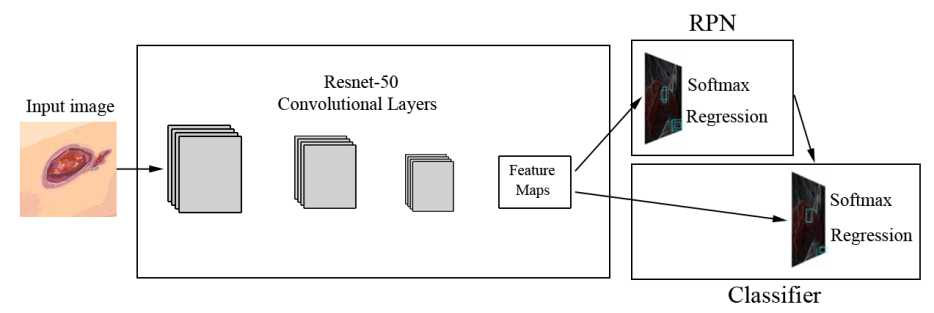


**Fig. s3** **Faster R-CNN for object detection of DFU ^[42]^** Images of DFU were input the adapted Faster R-CNN model based on Resnet-50 to obtain feature map. Proposals were generated and refined by using the feature map extracted. Region proposals were generated and refined by using the feature map. The images were classified and refinery precisely located.

.


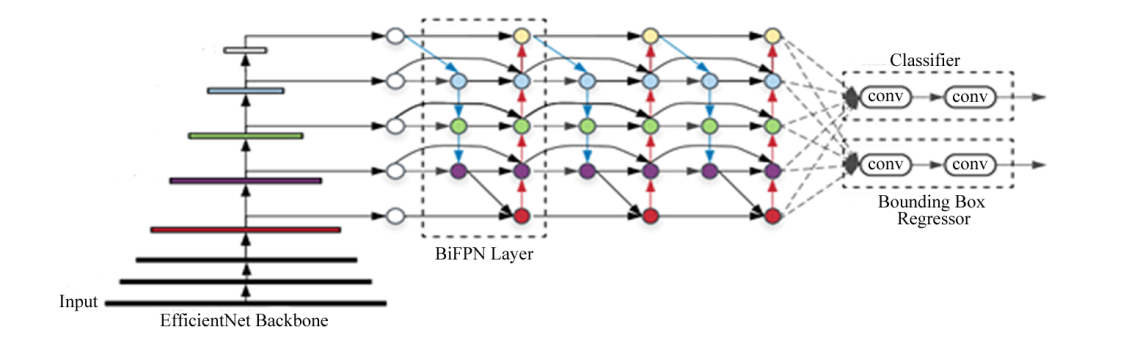


**Fig. s4** **Architecture of a** **EfficientNet for object detection^[35]^** The model uses EfficientNet as the backbone network, BiFPN as the feature network, and shared class/box prediction network. Both BiFPN layers and class/box net layers are repeated multiple times.


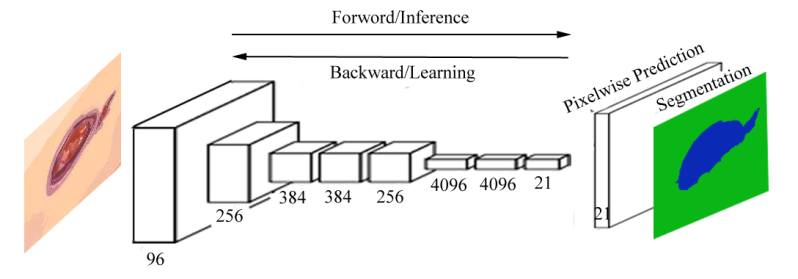


**Fig. s5 Architecture of fully convolutional networks for semantic segmentation ^[48]^** The model learned features with forword and backword learning for semantic segmentation.


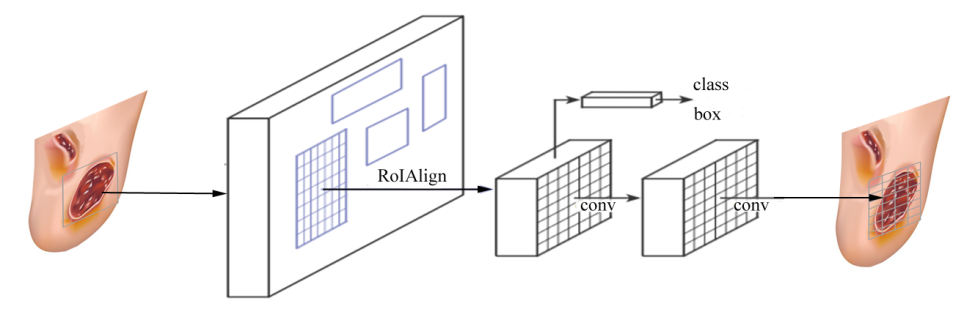


**Fig. s6 Mask R-CNN framework for** **instance segmentation ^[51]^**  Images of DFU are input the mask R-CNN model. The Mask-RCNN outputs an object mask, RoI boxes and bounding box regressor. This mask supports to do the object segmentation more accurately.

Note: According to the Wagner–Meggitt wound classification ^[16]^ for foot-ulcer evaluation, the images of DFU was grade 2 (deep ulcer to tendon, bone, or joint).


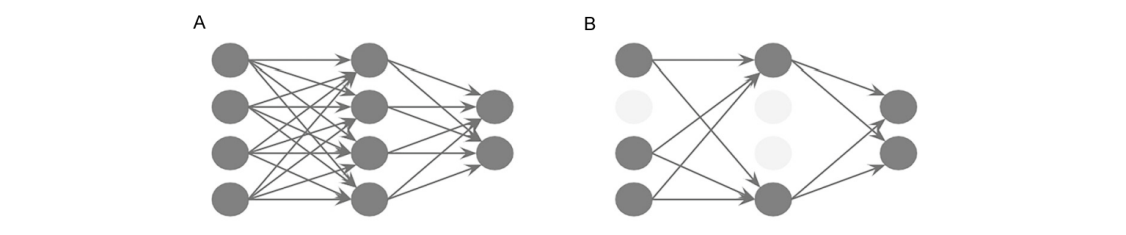


**Fig.s7 A conceptual view of dropout ^[57]^. Instead of using a fixed network structure, dropout randomly removes units from a fully connected network (A) to create a sub-model (B)**


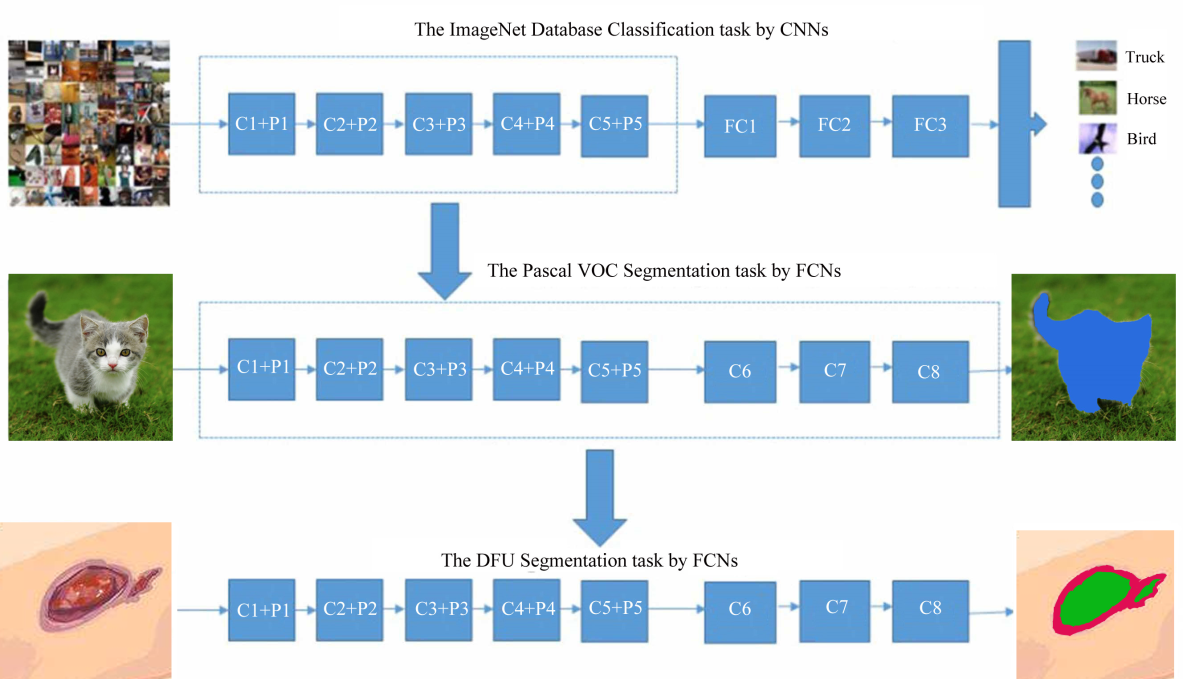


**Fig. s8 the two-tier transfer learning for the effective segmentation ^[52]^** Two-tier transfer learning were used. The CNN models are trained on the ImageNet dataset and the Pascal VOC segmentation dataset, respectively. These pre-trained models are used for training on DFU dataset to perform more effective segmentation on DFU dataset.

*Table S1 Confusion matrix*

| Confusion matrix | | Actual | |
| --- | --- | --- | --- |
|  |  | Positive | Negative |
| Predicted | Positive | TP | FP |
|  | Negative | FN | TN |
